# Remarks on the Cæsarean Section, Said to Have Been Performed on Jane Foster in 1793

**Published:** 1822-09

**Authors:** 

**Affiliations:** Surgeon, Wigan.


### Art. VIII.—*Remarks on the Cæsarean Section, said to have been performed on Jane Foster in* 1793. By ——— Hawarden, Surgeon, Wigan.

I trust you will give insertion to the following remarks connected with the account of what Mr. Barlow calls a case of Cæsarean operation, performed on Jane Forster, of Blackrod, in 1793.

In a note, he states that Mr. Thomas Hawarden of that place, but some years since deceased, was, at the beginning of the operation, suddenly seized with a violent fit of syncope which wholly prevented him from attending to the steps of the operation, and that he was obliged to be assisted by a female attendant. In justice to my brother's memory I will just repeat, 1st, his account of the operation, and 2dly, the deposition of the female attendant.

My brother, Mr. Thomas Hawarden, since dead, was at my house about ten days after the operation, when I requested him to describe it to me as correctly as he could. He stated to me, that the patient was laid on her back upon a blanket, with her head rather raised, on a table; and an incision made on the left side of the linea alba, about five or six inches long, from the navel, through the skin and membrana adiposa, and very cautiously afterwards through the peritoneum; which being done, a smooth, roundish substance presented itself, on which Mr. B., looking up, and addressing him, said "This is the uterus;" but, upon making an incision into it, about four inches long and half an inch deep, he then said "I believe it will prove the breech after all." The child was in contact with the bowels, and it and the placenta were brought away with the greatest ease, and no loss of blood. The wound was afterwards closed and sponged, and six or seven stitches taken through the integuments; but I never saw any part of the uterus, which, I apprehend, had been ruptured two days before, when the pains suddenly ceased." I here said, "It was, after all, then, not a case of Cæsarean operation, strictly speaking, but of extraction of extra-uterine fœtus?"—"Exactly so," was his reply.

Now follows the deposition of Catharine Clarkson.—She was resident at Blackrod, and about thirty-six years old at the time of the operation. My brother's widow found her at home, and brought her to another gentleman, a surgeon, and myself. I first asked her if my brother had fainted at the beginning of the operation, and had remained unable to assist during the remainder of the operation? "No such thing," she answered; "he was very busy all the time, and I threaded and gave him two crooked needles; and, after all was over, I brought him a bason of warm water and a towel."

So much for the syncope. To me it seems introduced to favour the story of the uterus and its unusual thinness, &c. But why were no sutures taken in it? In the two latter cases of the three, sutures were used, but both patients died.

*Wigan; July 6th, 1822.*

